# Quantification of Human Milk Phospholipids: the Effect of Gestational and Lactational Age on Phospholipid Composition

**DOI:** 10.3390/nu11020222

**Published:** 2019-01-22

**Authors:** Ida Emilie Ingvordsen Lindahl, Virginia M. Artegoitia, Eimear Downey, James A. O’Mahony, Carol-Anne O’Shea, C. Anthony Ryan, Alan L. Kelly, Hanne C. Bertram, Ulrik K. Sundekilde

**Affiliations:** 1 Department of Food Science, Aarhus University, 5792 Årslev, Denmark; idaemilielindahl@food.au.dk (I.E.I.L.); virginia.artegoitia@food.au.dk (V.M.A.); hannec.bertram@food.au.dk (H.C.B.); 2 School of Food and Nutritional Sciences, University College Cork, T12 YN60 Cork, Ireland; eimeardowney@gmail.com (E.D.); sa.omahony@ucc.ie (J.A.O.); a.kelly@ucc.ie (A.L.K.); 3 Department of Paediatrics and Child Health, University College Cork, T12 YN60 Cork, Ireland; Ca.OShea@ucc.ie (C.-A.O.); tony.ryan@ucc.ie (C.A.R.)

**Keywords:** Human Milk, Preterm infant, Phospholipids, Lipidomics, Milk Fat Globule Membrane

## Abstract

Human milk (HM) provides infants with macro- and micronutrients needed for growth and development. Milk phospholipids are important sources of bioactive components, such as long-chain polyunsaturated fatty acids (LC-PUFA) and choline, crucial for neural and visual development. Milk from mothers who have delivered prematurely (<37 weeks) might not meet the nutritional requirements for optimal development and growth. Using liquid chromatography tandem-mass spectrometry, 31 phospholipid (PL) species were quantified for colostrum (<5 days postpartum), transitional (≥5 days and ≤2 weeks) and mature milk (>2 weeks and ≤15 weeks) samples from mothers who had delivered preterm (*n* = 57) and term infants (*n* = 22), respectively. Both gestational age and age postpartum affected the PL composition of HM. Significantly higher concentrations (*p* < 0.05) of phosphatidylcholine (PC), sphingomyelin (SM) and total PL were found in preterm milk throughout lactation, as well as significantly higher concentrations (*p* < 0.002) of several phosphatidylethanolamine (PE), PC and SM species. Multivariate analysis revealed that PLs containing LC-PUFA contributed highly to the differences in the PL composition of preterm and term colostrum. Differences related to gestation decreased as the milk matured. Thus, gestational age may impact the PL content of colostrum, however this effect of gestation might subside in mature milk.

## 1. Introduction

Human milk (HM) is deemed the ideal choice when it comes to nutrition of infants and young children (<6 months of age). HM provides fat, carbohydrate, protein and micronutrients needed for postnatal growth and development, contributes to passive immunity of the infant and promotes healthy microbial colonization [1,2]. During the first six months postpartum, lipids make up 45–55% of the total energy content of HM, equivalent to a mean fat content of approximately 3.7–9.1 g/100 kcal [3,4]. Fatty acids (FA) in the form of triacylglycerol (TAG) constitutes the vast majority of milk lipids (< 98%) [5] and are emulsified in structures called milk fat globules (MFG). A distinct trilayer phospholipid (PL) membrane makes up the milk fat globule membrane (MFGM) and contributes to approximately 60% of the PLs found in HM, whereas the remainder is found as MFGM aggregates and PL assemblies termed extracellular vesicles in the skim milk fraction [6,7].

Albeit constituting a mere 1% of the total lipid content of milk [6], dietary PLs play an important role in digestion, absorption and transport of TAG [8]. The glycerophospholipids (GPL) phosphatidylcholine (PC) and phosphatidylethanolamine (PE) and the sphingolipid (SL) sphingomyelin (SM) are the three major PL classes found in HM. Provision of important bioactive components, for example, choline and long-chain polyunsaturated fatty acids (LC-PUFA) such as arachidonic acid (AA), eicosapentaenoic acid (EPA) and docosahexaenoic acid (DHA) is crucial for neural and visual development and growth of the neonate [9,10,11]. 

Days postpartum (lactational stage), gestational age and maternal diet are factors known to influence milk composition [12]. Several studies have investigated the macronutrient [13] and micronutrient [14] composition of milk from mothers who delivered prematurely and at term and suggested that preterm milk might not meet the nutritional requirements of the preterm infant, potentially increasing the risk of impaired growth and development. Metabolomic studies on preterm and term milk have furthermore revealed significant differences in metabolites important for infant development, including phosphocholine, citrate and lactose [15], cholesterol, saturated FA and monounsaturated FA [16] as well as glutamine and lysine [17].

Nonetheless, studies on the effect of PL concentration and composition, comparing preterm and term milk over the course of lactation, are scarce and total quantification of major PL species has not been performed. Thus, the objective of this study was to elucidate how age at gestation affects the PL composition and concentration of human colostrum and milk during the first 15 weeks postpartum, utilizing liquid chromatography tandem-mass spectrometry (LC-MS/MS).

## 2. Materials and Methods

### 2.1. Ethical Approval

All subjects gave their informed consent for inclusion before they participated in the study. The study was conducted in accordance with the Declaration of Helsinki and the protocol was approved by The Clinical Research Ethics Committee of the Cork Teaching Hospitals, Cork, Ireland (clinical number reference ECM 4(s) 06/08/13).

### 2.2. Chemicals and Reagents

The standards L-alpha-phosphatidylcholine (from egg yolk, ≥99%) and 3-sn-phosphatidylethanolamine (from bovine brain, ≥98%) were purchased from Sigma-Aldrich (St. Louis, MO, USA); The sphingomyelin (bovine buttermilk, >98%) standard was purchased from Larodan (Solna, Sweden). The SPLASH® LipidoMix™ deuterated internal standard (containing deuterated PC C15:0/C18:1, PE C15:0/18:1 and SM C15:0/18:1) was purchased from Avanti Polar Lipids Inc. (Birmingham, AL, USA). Mass spectrometry-grade ammonium formate (99.0%) and HPLC-grade chloroform (≥ 99.8%), acetonitrile and methanol were purchased from Sigma-Aldrich (St. Louis, MO, USA). LC-MS grade formic acid (98–100%) was purchased from Merck (Darmstadt, Germany). Preparation of ultrapure water was carried out using a water purification system from Holm & Halby (Brøndby, Denmark).

### 2.3. Preparation of Standards

Stock solutions of PC (100 mg/100 mL), PE (100 mg/100 mL) and SM (125 mg/100 mL) standards were prepared in chloroform and stored at −20 °C. For the calibration curve, a mixed solution containing all three standards was prepared in ice-cold methanol. The mixture solution was serial diluted in methanol to obtain eight dilutions of the following standard concentrations; PC 0.2–25 mg/100 mL; PE 0.1–13 mg/100 mL; and SM 0.1–15.8 mg/100 mL. To each of the eight dilutions was added 1 µL internal standard mix. High and low quality controls (QC) were constructed based on the mixture solution, to control drift and deterioration of the system. High QC were constructed with 6.3 mg/100 mL PC, 3.3 mg/100 mL PE and 4.0 mg/100 mL SM; Low QC were constructed with 0.2 mg/100 mL PC, 0.1 mg/100 mL PE and 0.1 mg/100 mL SM.

### 2.4. Milk Sample Collection

Preterm and term colostrum and milk samples of known gestational age and lactational stage were collected from non-smoking, non-diabetic mothers as described earlier [15]. Briefly, preterm HM samples were provided by the neonatal intensive care unit of Cork University Maternity Hospital (Wilton, Co., Cork, Ireland). Colostrum and HM samples (*n* = 57) spanning lactation from 1 day to 15 weeks postpartum were donated by 14 mothers who had delivered healthy, preterm (delivered before the 37th week of gestation) infants. The mean age of the mothers was 34 ± 5.3 years, mean gestational age was 30.8 ± 3.4 weeks and mean infant weight at birth was 2064 ± 847.2 grams. 

Pasteurized and microbiologically screened term (delivered from the 37th week of gestation) milk samples were provided by The Western Trust Milk Bank (Irvinestown, Co., Fermanagh, Ireland). These samples (*n* = 22) were donated by 22 mothers, spanning stages of lactation from <5 days postpartum to 15 weeks postpartum. Preterm and term samples were kept at −80 °C until analysis. 

### 2.5. Liquid-Liquid Extraction of Phospholipids

Extraction of polar lipids from technical replicates was carried out in random order, creating *n* = 158 HM samples. A Modified Bligh and Dyer protocol was used for PL extraction [18]. Milk samples were thawed on ice prior to extraction, followed by 40 seconds of homogenization by vortexing. In a centrifuge tube, 70 µL of milk was mixed with 1 µL of deuterated internal standard and vortexed briefly. To the centrifuge tube, 1 mL of ice-cold chloroform:methanol:water (1:2:0.8) was added and vortexed for 20 seconds, followed by centrifugation at 4 °C for 10 minutes at 20,000 g. In a new centrifuge tube, the organic and aqueous phases were transferred and the solvents evaporated at 30 °C. One mL ice cold methanol was added for resuspension of the dried lipids and proteins were precipitated by incubation at −20 °C for 30 minutes. Centrifugation at 20,000 g for 10 minutes at 4 °C was followed by transfer of the supernatant to a new centrifuge tube. Until analysis, the lipid extracts were stored at −80 °C. Prior to analysis, centrifugation of the lipid extract was carried out at 20,000 g for 10 minutes at 4 °C to precipitate residual proteins, followed by transfer of 200 µL of lipid extract to glass vials.

### 2.6. Analysis of Phospholipid Species

An established LC-MS method for quantification of PL species (mg/100 mL) was based on a method described by Zhao et al. [19]. Sample analysis was performed on an Agilent 1290 Ultra Performance Liquid Chromatography system coupled to an Agilent 6495 Triple Quadrupole Mass Spectrometer (Agilent Technologies, Palo Alto, CA, USA). The LC was equipped with an autosampler (kept at 4 °C) and the MS with an ESI source. Chromatographic separation was conducted on a Waters HILIC column (2.1 × 100 mm with particle size 1.7 µm) protected by a Waters HILIC guard column (2.1 mm × 5 mm) (Waters Corporation, Milford, MA, USA). The column oven temperature was set to 25 °C. The LC conditions consisted of water with 10 mM ammonium formate (eluent A) at pH 3 (adjusted with formic acid) and acetonitrile (eluent B). The column was equilibrated prior to chromatographic separation. The following gradient was applied for separation of analytes: the mobile phase was held constant at 95% eluent B from 0 to 0.10 min; from 0.l0 to 20 min the gradient decreased to 80% eluent B; from 20 to 21 min the gradient decreased to 70% eluent B, after which the mobile phase was held constant from 21 to 23 min; from 23 to 24 min the mobile phase was increased to 95% eluent B, followed by equilibration of the column at 95% eluent B from 24 to 26 min. An injection volume of 5 µL was used. The ESI ion source (operated in positive mode) and MS were operated under the following conditions: capillary voltage 4500 V, nebulizer 1000 V, temperature of the source 210 °C, cone gas (ultrahigh-purity nitrogen) 17 L/min, desolvation temperature and sheath gas temperatures of 230 °C and 400 °C, respectively, both with gas flow rates of 11 L/h. High and low funnel radio frequencies were 200 eV and 160 eV, respectively. PE, PC and SM PL standards were used to develop a dynamic multiple reaction monitoring (MRM) method for the MS. Internal standards added during sample preparation were used for the absolute quantification of PLs. 

### 2.7. Preprocessing of Data and Statistical Analysis

For retrieval and pre-processing of LC-MS data, the Agilent MassHunter Workstation Software -Data Acquisition for 6400 Series Triple Quadrupole program version B.07.00 was used (Agilent Technologies Inc., Santa Clara, CA, USA). Chromatograms were manually inspected and fitted when needed. Calibration curves were constructed containing at minimum of five points. A regression coefficient cut-off of ≥0.95 was used. Multivariate exploratory data analysis was carried out using SIMCA version 15.0.2 (Sartorius Stedim Biotech, Umeå, Sweden) to identify clustering as well as used for outlier detection. Unit variance scaling was conducted prior to analysis. Minitab version 18 (Minitab Inc., Pennsylvania, United States) was used for analysis of variance (ANOVA) utilizing a two-way factorial comparison of PL classes and -species as the dependent variable(s), with gestational age and lactational stage as independent variables. A p-value of *p* < 0.05 was considered significant for testing of PL classes. For the multiple testing of PL species, a corrected significance level of *p* < 0.002 was calculated using Bonferroni correction. Tukey’s test was used as a post-hoc test for multiple comparisons of means, using upper- and lower-case letters to illustrate significance related to lactational stage and gestational age, respectively. In case of statistical interaction, that is, if either of the main factors were dependent on the other, the superscript “i” was used for annotation purposes.

## 3. Results

### 3.1. Percentage Distribution of Phospholipids

**Figure 1:**
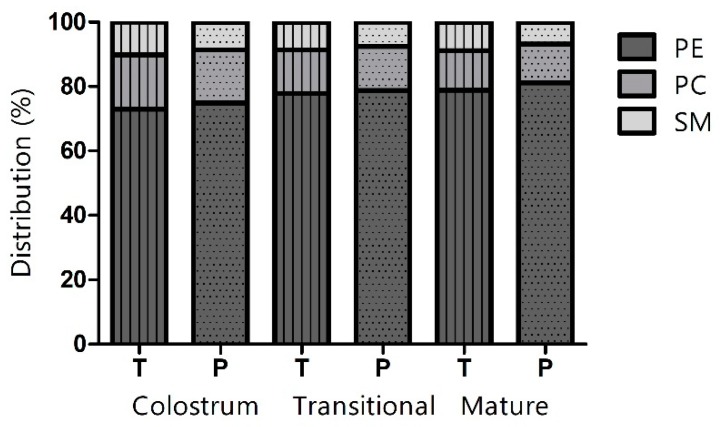
Percentage distribution of phosphatidylethanolamine (PE), phosphatidylcholine (PC) and sphingomyelin (SM) in term (T; vertically striped bars) and preterm (P; dotted bars) colostrum, transitional and mature milk.

Identification and quantification of 31 PL species were achieved by LC-MS/MS. Milk samples were divided into three lactational stages depending on days postpartum, that is, colostrum (<5 days), transitional milk (≥ 5 days and ≤2 weeks) and mature milk (> 2 weeks and ≤15 weeks), as well as divided according to gestational age, that is, preterm milk and term milk. Figure 1 illustrates the percentage distribution of the three PL classes. The majority of the PLs were constituted by PE, followed by PC and SM, in both preterm and term colostrum, transitional and mature milk.

In both preterm and term milk, interaction (*p* < 0.05) was observed between gestational age and lactational stage for all three PL classes; nevertheless, the percentage of PE was observed to increase from colostrum to mature milk (*p* < 0.05), whereas the percentage of PC decreased (*p* < 0.05). The percentage constituted by SM in preterm milk decreased from colostrum to mature milk, whereas the percentage of SM in term milk showed a decrease from colostrum to transitional milk, followed by a slight increase (*p* < 0.05). 

### 3.2. Total Concentration of Phospholipids

The concentration (mg/100 mL) of total PLs in preterm and term milk at three lactational stages (colostrum, transitional and mature milk) is shown in Table 1. The concentration of total PLs in both preterm and term milk was found to decrease significantly (*p* < 0.05) with progression from colostrum to transitional milk and from transitional milk to mature milk. Furthermore, the concentration of total PLs was significantly higher in preterm milk compared to term milk throughout lactation (*p* < 0.05). The same pattern of significance was observed for the PL classes SM and PC. No significant differences in the concentration of PE were observed between colostrum and transitional milk in both preterm and term milk. 

### 3.3. Concentrations of Individual Phospholipid Species

A total of 31 individual PL species from three different PL classes were quantified in preterm and term milk at three lactational stages (Table 2). The concentrations of the majority of the PL species were found to be significantly affected by both gestational age of the neonate and lactational stage postpartum. In general, preterm milk exhibited higher concentrations of individual PL species compared to term milk. The concentration of the individual PL species also decreased with time postpartum. 

Significant differences (*p* < 0.002) in the concentration of PL species were found in the earlier stages of lactation. A decrease in the concentration of the SM species C18:1/C20:0, C18:0/C22:1 and C18:0/C22:0 as well as the PC species C16:0/C18:2 and C16:0/C18:1 was observed between colostrum and transitional milk in both preterm and term milk, with higher concentrations in preterm milk compared to term milk. 

Effects throughout the entire lactational period were observed related to the SM species C18:0/C20:0, the PC species C16:0/C16:0 and C16:0/C16:1 and the PE species C18:1/C16:0 and C18:1/C18:0. A significant decrease (*p* < 0.002) in their concentrations were observed from colostrum to transitional milk, as well as from transitional to mature milk, in both preterm and term milk. No significant differences were observed between colostrum and transitional milk with regards to the concentration of PE species C18:0/C22:6 and C18:0/C16:0; however, a significant decrease (*p* < 0.002) was observed in mature milk compared to colostrum and transitional milk. Moreover, for the PE species C18:0/C18:1, C16:0/C22:6 and C16:0/C20:4 and the PC species C18:0/C18:2, C16:0/C20:5 and C16:0/C20:3, no significant differences were observed between colostrum and transitional milk, as well as between transitional milk and colostrum. However, the species decreased significantly (*p* < 0.002) from colostrum to mature milk. Finally, preterm milk contained significantly higher (*p* < 0.002) concentrations of all of the aforementioned PL species compared to term milk throughout lactation.

Neonate age at parturition alone also showed to affect the concentration of several PLs, as the concentration of the SM species C18:1/C14:0, C18:0/C24:0 and C18:0/C14:0 and the PE species C16:0/C18:1, C16:0/C18:2, C18:0/C18:2 and C18:1/C18:1 were all significantly higher (*p* < 0.002) in preterm milk compared to term milk. 

### 3.4. Exploratory Analysis of Preterm and Term Milk

**Figure 2:**
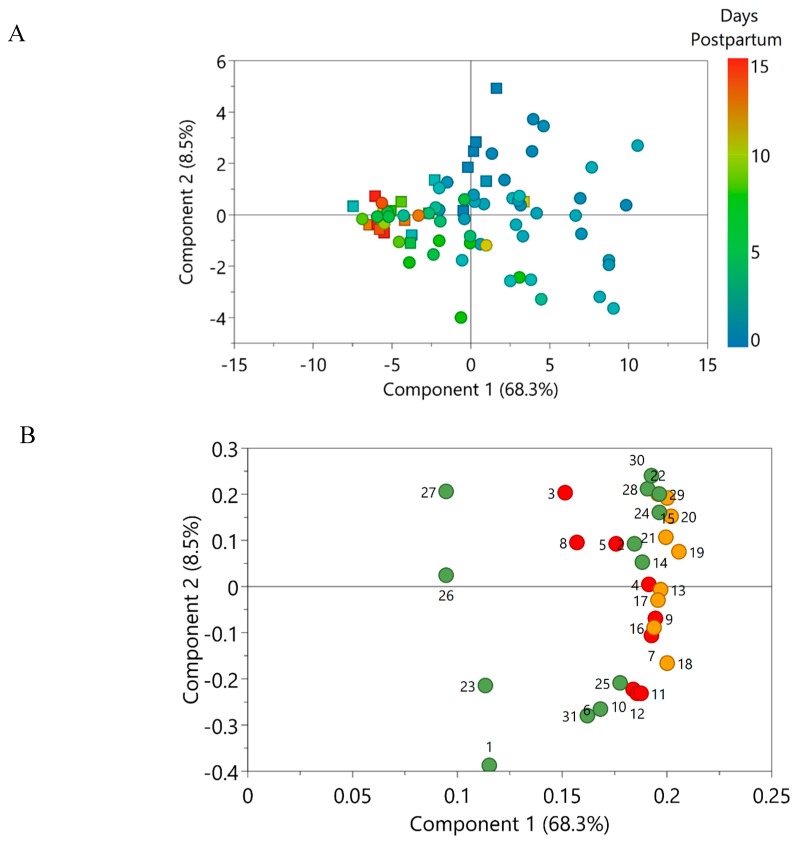
(**A**) PCA score scatter plot of preterm milk (squares) and term milk (circles) expressed between day 1 and 15 weeks post-partum. Score colours indicate day postpartum at which the samples were expressed. (**B**) Corresponding loading scatter plot containing the 31 phospholipid species quantified in human milk belonging to the phospholipid classes phosphatidylethanolamine (red circles), phosphatidylcholine (orange circles) and sphingomyelin (green circles). Details about the numbering of phospholipid species can be found in Table 2.

**Figure 3:**
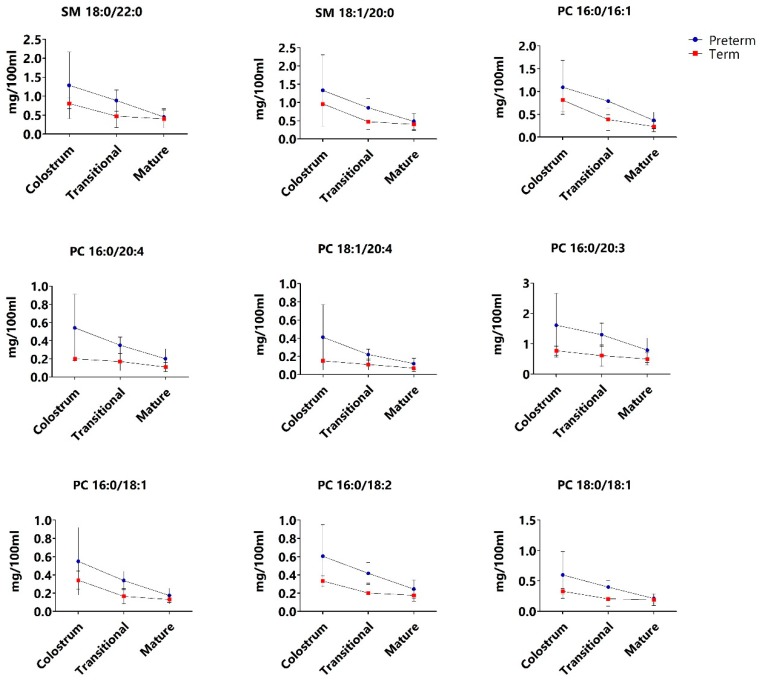
Concentration (mg/100 mL) of phosphatidylcholine (PC) and sphingomyelin (SM) species in preterm (blue circle) and term (red square) milk in colostrum (<5 days), transitional milk (≥5 days and ≤2 weeks) and mature milk (>2 weeks and ≤15 weeks).

Exploratory multivariate data analysis was used to evaluate the changes in PL composition with progression of lactation in preterm and term milk. A Principal Component Analysis (PCA) model was constructed and principal components 1 and 2 collectively explained 76.8% of the variance. The accompanying score scatter plot (Figure 2A) showed a clear progression with lactation along the first principal component, whereas the separation of preterm and term colostrum was evident on both first and second principal components. Furthermore, grouping of preterm and term milk became more pronounced with progression from transitional milk to mature milk. These findings suggest that the PL composition of both preterm and term milk changes with progression of lactation. In addition, differences in the composition of PLs between preterm and term milk became less apparent as lactation progressed. The loadings scatter plot (Figure 2B) furthermore substantiated that the observed variance was associated with a higher content of PLs in early lactation; thereafter, differences in PL content in preterm and term milk subsided with progression of lactation. The PL species that contributed the most to the variance are presented in Figure 3.

Based on the exploratory analysis (Figure 2), PL species from the PC class was found to strongly contribute to the differences observed between preterm and term milk throughout lactation. There was large variability in preterm and term colostrum with regards to the concentration of two SM species and several PC species (Figure 3), nearly all of which contained long-chain polyunsaturated fatty acids (LC-PUFA). These differences diminished with time postpartum as the concentration decreased in both preterm and term milk (albeit to a higher extend in the former), resulting in discrete differences in the concentrations of these PLs in mature preterm and term milk.

## 4. Discussion

In general, the PL content of HM was found to be influenced by the age of the infant at parturition as well as days postpartum. Throughout lactation, preterm colostrum and milk were found to contain significantly higher concentrations of total PLs, as well as several PL species and -classes, compared to term colostrum and milk. The differences observed in PL composition of preterm and term colostrum were particularly influenced by differences in the concentration of PC species containing LC-PUFA moieties. The observed differences between preterm and term colostrum were less prominent in mature milk. 

The percentage distribution of PLs in term HM have been studied by several authors [9,20,21,22] but there are significant discrepancies between studies, as previously reported [12]. In the present study, regardless of gestational age, PE represented the largest proportion of PLs throughout lactation, followed by PC and SM. These findings deviate from findings by Bitman et al. [21] and Benoit et al. [22], who reported SM to constitute the largest proportion of PLs in HM (36% [21]; 43.3% [22]), followed by PE (20% [21], 21.3% [22]) and PC (28% [21], 19.0% [22]). In contrast, a study by Sala-Vila et al. [20] reported even larger differences in the PL distribution of term milk, ascribing SM and PC to contribute the vast majority of PLs (39.2–41.0% and 31.3–38.4%, respectively), while PE only contributed with a minor proportion (5.9–12.76%). Nonetheless, Wang et al. [9] reported PE to be the major PL contributor (~36.1%) in term milk, followed by SM (~30.7%) and PC (~23.1%). Our findings suggest that the proportion of PE increased with progression of lactation in both preterm and term milk; however, this main effect was dependent on gestational age. Nonetheless, findings by Sala-Vila et al. [20] showed that the proportion of PE increased significantly from colostrum to transitional milk and from transitional milk to mature milk.

To our knowledge, absolute quantification of individual PL species in preterm milk over the course of lactation has not been published previously. Independent of gestational age, the concentration of total PLs quantified was found to be the highest in colostrum. In addition, the concentration of total PLs decreased with progressing lactation for both preterm and term milk. This is in accordance with the report of Bitman et al. [21] of a decrease in relative PL content from colostrum to mature milk. Additionally, higher contents of PLs throughout lactation in very preterm milk (delivered between the 26–30th gestational week) and preterm milk compared to term milk, were reported [21]. The measured concentrations of PC and SM in term milk over the course of lactation are in agreement with Claumarchirant et al. [23] and Ma et al. [24]. Conversely, the concentration of PE contributed heavily to the discrepancies between this and other studies. The concentrations found in this study was 49.64 ± 13.68 mg/100 mL in term colostrum and 29.15 ± 13.04 mg/100 mL in term mature milk (≤15 weeks postpartum). In contrast, Claumarchirant et al. [23] found the concentration of PE to range between 15.9-9.5 mg/100 mL, while the concentrations of PE measured by Ma et al. [24] was 9.0 ± 2.6 mg/100 mL in colostrum and 3.9 ± 1.6 mg/100 mL in mature milk.

Discrepancies between the present study and other studies might stem from the diverse nature of PLs. GPL consist of a glycerol backbone, to which fatty acyls are bound to the first and/or second carbon and a phosphate group with a polar headgroup attached to the third carbon; accordingly, the SL SM consists of a sphingosine backbone with phosphocholine and a fatty acyl attached [25]. The structural composition of both GPL and SL enables an array of individual PL species to be found within a PL class, depending on the attached fatty acid moieties. Hence, the discrepancies between studies might originate from the fact that measurements are conducted on different PL species. Furthermore, the majority of studies on the PL composition of HM do not report on the individual PL species but rather the distribution of individual fatty acyl moieties contributing to the various PL classes, thus precluding direct comparisons between studies. Discrepancies might also be influenced by the use of an internal standard during sample preparation. In the present study, the method used for quantification of PLs included the addition of deuterated internal standards prior to PL extraction. Addition of an internal standard is carried out to correct for potential analyte losses or analytical errors during sample preparation [26] and similarly during MS acquisition, which, along with the use of dynamic MRM, strengthens the quantification performed in this study. This approach might also explain the higher concentrations of PE observed in this study. In contrast, the study by Ma et al. [24] employed external standards for method validation.

Besides quantification of the three major PL classes in HM, total lipid content of the preterm and term milk samples from the present study was furthermore analysed by Guerra et al. [16] using GC-MS. To summarize, the mean concentration of total lipids in the preterm milk samples was significantly higher than the term milk samples [16]. Lactational stage also affected total lipid content, as the lipid concentration increased with maturation of the milk [16]. This inverse relationship between total lipid content and PLs is in agreement with finding by others [27]. As the milk matures, TAG synthesis in the ER of the alveolar cells increases, along with the demand for membrane material for the formation of cytoplasmic lipid droplets (CLD). As a result of the limited availability of PLs, coalescence of CLD can relieve the demand for PLs, subsequently leading to the release of larger MFGs into the lumen. 

In preterm milk, both the total PL and total lipid concentration decreased with progression of lactation, which is in contrast to the findings in term milk [16]. These findings might indicate an altered MFG size distribution and/or concentration of MFGs in preterm milk during early lactation. Differences in the size distribution of fat globules in preterm and term milk was also observed by Simonin et al [28], who found a higher content of small milk fat globules (SMFG) and large milk fat globules (LMFG) in preterm milk, as well as differences in the concentration of MFGs. These differences were observed to be more pronounced in colostrum and diminished in mature milk [28].

For hydrolysis and digestion of the globules in the infant gastrointestinal tract, an increased surface area for anchoring of gastric lipase [29] would be the result of a higher SMFG fraction in preterm colostrum and milk, which might subsequently be of benefit due to the immature gastrointestinal tract and reduced lipolytic capacity of premature infants. A higher PL content have previously been related to the SMFG fraction in bovine milk [30].

Independent of gestation, PLs containing the LC-PUFA AA and DHA were especially prevalent in PE, which is in accordance with previous studies, that reported that DHA and EPA (an intermediate of DHA synthesis) were more abundant in PE than PC and SM [9]. DHA and AA are often found as FA moieties of PE and PC and contribute to myelination, as well as neurological and visual development of the neonate [31,32].

The concentration of 25 out of 31 individual PL species were found to be higher in preterm milk compared to term milk throughout lactation. Interestingly, multivariate analysis showed that the PL species which contributed the most to the differences in preterm and term milk early postpartum belonged to the PC and SM class. PC and SM are both important sources of choline, as they account for approximately 17% of choline in HM [12]. Choline is also found in HM as free choline, phosphocholine and glycerophosphocholine. Accretion of choline begins in utero, as choline is transferred from the placenta to the foetus, playing crucial roles in organ development of, for example, the brain and neural tube [33], in membrane biosynthesis [12] and in normal cell function [34]. The importance of choline for infant development continues postpartum and includes involvement in the structural integrity of cell membranes [35], as a precursor for acetylcholine in cholinergic neurotransmission [11] and as a methyl-donor for the synthesis of methionine [34]. Studies have furthermore suggested increased choline requirements of preterm infants [34]. 

Besides palmitic acid (C16:0), the LC-PUFA AA (C20:4), DGLA (C20:3) and linoleic acid (C18:2) from the N6-series of FAs were the principal FA that were attached to the PC species that contributed to the observed differences in preterm and term PLs early postpartum. The accretion of these LC-PUFA is likewise involved in neural and visual development and they are accumulated in the foetus during the last trimester [20].

The altered PL composition of preterm colostrum and milk subsided with maturation and our findings indicate that the composition of mature (3–15 weeks) preterm milk might be similar to that of mature term milk. A similar trend was observed by Sundekilde et al. [15] for the metabolite profiles of the preterm and term colostrum and milk samples analysed in the present study. By proton NMR spectroscopy, the metabolome of preterm colostrum was revealed to differ from that of term colostrum, after which preterm milk changed with progression of lactation, which resulted in the composition of preterm milk resembling term milk at 5–7 weeks postpartum [15]. Other milk constituents such as carbohydrates, fat [36], protein and minerals [37] have similarly been reported to be affected by gestational age. Preterm infants are delivered when the in utero growth-rate is at its highest [38]; thus, the growth-rate of preterm infants is much higher than for infants delivered at term [39].

The composition of HM, especially the lipid fraction, is known to be subject to both inter- and intraindividual variability [3,40]. For the concentration of PL in both preterm and term samples, large standard deviations were calculated. Diurnal variation, variation during a feed (foremilk or hindmilk), as well as maternal diet, are factors known to affect the lipid fraction [41]. The mentioned factors were uncontrolled in this study, which could possibly have contributed to the observed variations. The small sample size is a substantial limitation to this study and corroboration of the work presented is therefore necessary.

## 5. Conclusions

In this study, the effect of time postpartum and gestational age on the composition of the three major PL classes in milk (PE, PC and SM) constituting a total of 31 PL species, was evaluated. The concentration of PC and SM as well as total PL was significantly higher in milk from preterm mothers throughout lactation. Accordingly, the majority of PL species from all three PL classes were significantly higher in preterm milk. The observed differences between preterm and term PL composition were most pronounced in colostrum and attenuated in mature milk. However, due to the small sample size, confirmation of these findings is needed.

The underlying mechanisms behind the alterations of early lactation PL composition of preterm milk, are unknown. However, immaturity of the mammary and/or a compensatory mechanism to meet the higher metabolic demands in preterm infants gland might influence the observed phenotype [42]. Nevertheless, advancements must be made in the understanding of how to adequately meet the nutritional needs of preterm infants, to ensure preterm growth and development on par with infants delivered at term. 

## Figures and Tables

**Table 1:** Concentration of phospholipids (mg/100 mL) in colostrum, transitional and mature milk from mothers with preterm and term gestations.

Phospholipid Class	Preterm						Term					
	Colostrum		Transitional		Mature		Colostrum		Transitional		Mature	
PE	87.85	(27.30) ^A,a^	75.25	(24.85) ^A,a^	46.64	(19.81) ^B,a^	49.40	(13.68) ^A,a^	37.86	(14.00) ^A,a^	29.15	(13.04) ^B,a^
PC	19.31	(11.46) ^A,a^	13.09	(3.94) ^B,a^	6.92	(3.23) ^C,a^	11.44	(2.64) ^A,b^	6.56	(3.26) ^B,b^	4.50	(1.97) ^C,b^
SM	10.13	(6.24) ^A,a^	7.21	(1.95) ^B,a^	3.93	(1.63) ^C,a^	6.90	(1.26) ^A,b^	4.23	(1.88) ^B,b^	3.29	(1.73) ^C,b^
Total PL	117.30	(42.08) ^A,a^	95.49	(29.43) ^B,a^	57.49	(23.77) ^C,a^	67.74	(14.47) ^A,b^	48.65	(18.11) ^B,b^	36.94	(16.41) ^C,b^

Two-way ANOVA with Tukey’s test of concentration means and standard deviations (in brackets). PE: Phosphatidylethanolamine, PC: Phosphatidylcholine, SM: Sphingomyelin, PL: phospholipid. Values with different superscript letters are significantly different (*p* < 0.05) within a row (upper case letters A, B, C indicate significant differences related to lactational stage; lower case letters a, b indicate significant differences related to gestational age).

**Table 2:** Concentration (mg/100 mL) of phospholipid species in colostrum, transitional and mature milk from mothers with preterm and term gestations.

#	Phospholipid Species	Preterm						Term					
		Colostrum		Transitional		Mature		Colostrum		Transitional		Mature	
1	PE (18:1/18:1)	0.78	(0.47) ^a^	1.09	(0.63) ^a^	0.92	(0.54) ^a^	0.43	(0.26) ^b^	0.38	(0.15) ^b^	0.52	(0.39) ^b^
2	PE (18:1/18:0)	>12.00	(5.80) ^A,a^	>12.00	(5.53) ^B,a^	6.66	(3.28) ^C,a^	12.00	(4.67) ^A,b^	6.58	(3.02) ^B,b^	3.52	(2.10) ^C,b^
3	PE (18:1/16:0)	7.55	(2.88) ^A,a^	4.63	(2.03) ^B,a^	2.79	(1.58) ^C,a^	4.46	(2.36) ^A,b^	2.76	(0.58) ^B,b^	1.56	(0.85) ^C,b^
4	PE (18:0/22:6)	7.45	(3.64) ^A,a^	7.94	(3.55) ^A,a^	3.57	(2.44) ^B,a^	3.60	(1.21) ^A,b^	3.05	(1.64) ^A,b^	1.81	(1.34) ^B,b^
5	PE (18:0/20:4)	6.43	(1.97) ^i^	4.09	(1.19) ^ii^	2.79	(1.13) ^iii^	3.05	(1.04) ^ii,iii^	2.58	(1.46) ^ii,iii^	2.13	(0.96) ^iii^
6	PE (18:0/18:2)	6.88	(3.13) ^a^	7.20	(2.54) ^a^	5.80	(2.56) ^a^	3.86	(1.42) ^b^	4.24	(2.50) ^b^	4.18	(1.82) ^b^
7	PE (18:0/18:1)	2.51	(1.11) ^A,a^	2.49	(0.85) ^AB,a^	1.50	(0.66) ^B,a^	1.46	(0.36) ^A,b^	1.18	(0.58) ^AB,b^	1.19	(0.64) ^B,b^
8	PE (18:0/16:0)	10.16	(3.50) ^A,a^	7.69	(3.57) ^A,a^	4.40	(2.07) ^B,a^	5.43	(2.25) ^A,b^	4.71	(1.17) ^A,b^	2.86	(1.26) ^B,b^
9	PE (16:0/22:6)	4.99	(2.15) ^A,a^	4.34	(1.87) ^AB,a^	2.42	(1.64) ^B,a^	2.24	(0.63) ^A,b^	1.57	(0.77) ^AB,b^	1.02	(0.63) ^B,b^
10	PE (16:0/20:4)	>12.00	(6.97) ^A,a^	11.78	(4.07) ^AB,a^	8.43	(3.97) ^B,a^	6.57	(2.37) ^A,b^	5.31	(2.46) ^AB,b^	4.94	(1.83) ^B,b^
11	PE (16:0/18:2)	9.00	(3.58) ^a^	9.11	(3.32) ^a^	6.12	(3.14) ^a^	5.07	(1.31) ^b^	4.50	(2.24) ^b^	4.52	(2.21) ^b^
12	PE (16:0/18:1)	1.99	(1.13) ^a^	2.30	(1.14) ^a^	1.25	(0.91) ^a^	1.23	(0.31) ^b^	1.01	(0.55) ^b^	0.90	(0.57) ^b^
13	PC (18:1/20:4)	0.41	(0.36) ^i^	0.22	(0.06) ^ii^	0.12	(0.06) ^iii^	0.15	(0.02) ^ii,iii^	0.11	(0.06) ^ii,iii^	0.07	(0.04) ^iii^
14	PC (18:0/18:2)	1.25	(0.68) ^A,a^	1.01	(0.27) ^AB,a^	0.66	(0.25) ^B,a^	0.67	(0.13) ^A,b^	0.63	(0.41) ^AB,b^	0.57	(0.29) ^B,b^
15	PC (18:0/18:1)	0.60	(0.38) ^i^	0.40	(0.11) ^ii^	0.21	(0.08) ^iii^	0.33	(0.05) ^ii^	0.20	(0.12) ^ii,iii^	0.19	(0.10) ^iii^
16	PC (16:0/20:5)	1.97	(1.46) ^A,a^	1.42	(0.47) ^AB,a^	0.80	(0.49) ^B,a^	0.76	(0.13) ^A,b^	0.64	(0.33) ^AB,b^	0.46	(0.26) ^B,b^
17	PC (16:0/20:4)	0.54	(0.37) ^i^	0.35	(0.09) ^ii^	0.20	(0.11) ^iii^	0.20	(0.02) ^ii,iii^	0.17	(0.10) ^ii,iii^	0.11	(0.05) ^iii^
18	PC (16:0/20:3)	1.61	(1.05) ^A,a^	1.30	(0.38) ^AB,a^	0.79	(0.40) ^B,a^	0.77	(0.15) ^A,b^	0.61	(0.35) ^AB,b^	0.50	(0.20) ^B,b^
19	PC (16:0/18:2)	0.61	(0.34) ^A,a^	0.42	(0.12) ^B,a^	0.25	(0.10) ^B,a^	0.33	(0.05) ^A,b^	0.20	(0.11) ^B,b^	0.18	(0.06) ^B,b^
20	PC (16:0/18:1)	0.55	(0.37) ^A,a^	0.34	(0.10) ^B,a^	0.18	(0.08) ^B,a^	0.34	(0.10) ^A,b^	0.17	(0.08) ^B,b^	0.13	(0.05) ^B,b^
21	PC (16:0/16:1)	1.09	(0.59) ^A,a^	0.79	(0.30) ^B,a^	0.37	(0.18) ^C,a^	0.81	(0.25) ^A,b^	0.38	(0.24) ^B,b^	0.23	(0.11) ^C,b^
22	PC (16:0/16:0)	10.68	(6.06) ^A,a^	6.78	(2.38) ^B,a^	3.36	(1.66) ^C,a^	7.06	(2.29) ^A,b^	3.44	(1.72) ^B,b^	2.06	(0.94) ^C,b^
23	SM (18:1/23:0)	0.22	(0.16)	0.19	(0.13)	0.15	(0.14)	0.13	(0.09)	0.12	(0.07)	0.09	(0.06)
24	SM (18:1/20:0)	1.33	(0.98) ^A,a^	0.85	(0.25) ^B,a^	0.48	(0.22) ^B,a^	0.96	(0.26) ^A,b^	0.47	(0.21) ^B,b^	0.40	(0.17) ^B,b^
25	SM (18:1/14:0)	0.52	(0.28) ^a^	0.56	(0.27) ^a^	0.31	(0.16) ^a^	0.36	(0.09) ^b^	0.21	(0.02) ^b^	0.23	(0.14) ^b^
26	SM (18:0/24:0)	0.08	(0.08) ^a^	0.04	(0.04) ^a^	0.03	(0.03) ^a^	0.02	(0.01) ^b^	0.01	(0.01) ^b^	0.02	(0.02) ^b^
27	SM (18:0/23:0)	0.02	(0.05)	0.01	(0.01)	0.01	(0.01)	0.02	(0.01)	0.01	(0.004)	0.01	(0.005)
28	SM (18:0/22:1)	1.35	(0.96) ^A,a^	0.90	(0.24) ^B,a^	0.52	(0.19) ^B,a^	0.85	(0.11) ^A,b^	0.52	(0.28) ^B,b^	0.51	(0.28) ^B,b^
29	SM (18:0/22:0)	1.29	(0.88) ^A,a^	0.89	(0.28) ^B,a^	0.45	(0.22) ^B,a^	0.81	(0.14) ^A,b^	0.47	(0.30) ^B,b^	0.40	(0.23) ^B,b^
30	SM (18:0/20:0)	5.02	(2.99) ^A,a^	3.39	(0.93) ^B,a^	1.78	(0.76) ^C,a^	3.56	(0.71) ^A,b^	2.27	(1.10) ^B,b^	1.47	(0.83) ^C,b^
31	SM (18:0/14:0)	0.29	(0.17) ^a^	0.38	(0.20) ^a^	0.22	(0.11) ^a^	0.19	(0.05) ^b^	0.15	(0.02) ^b^	0.17	(0.09) ^b^

Two-way ANOVA with Tukey’s test of concentration means and standard deviations (in brackets). #: Numbering of phospholipid species. PE: Phosphatidylethanolamine, PC: Phosphatidylcholine, SM: Sphingomyelin. Fatty acid moieties are indicated in parentheses. Values with different superscript letters are significantly different (*p* < 0.002) within a row (upper case letters A, B, C indicate significant differences related to lactational stage; lower case letters a, b indicate significant differences related to gestational age; roman letters i, ii, iii indicate significant differences related to interaction). The significance level has been adjusted using Bonferroni correction.
